# The correlates and experiences of HIV-related intersectional stigma among caregivers of adolescents living with HIV during COVID-19 in KwaZulu-Natal, South Africa: Results from a mixed method study

**DOI:** 10.1371/journal.pgph.0004296

**Published:** 2026-04-29

**Authors:** Nokwanda Sithole, Eugene Lee Davids, Zibuyisile Mkhwanazi, Stanley Carries, Lovemore Sigwadhi, Audrey Moyo, Darshini Govindasamy

**Affiliations:** 1 Health Systems Research Unit, South African Medical Research Council (SAMRC), Durban, South Africa; 2 Department of Psychology, Faculty of Humanities, University of Pretoria, Pretoria, South Africa; 3 Division of Epidemiology and Biostatistics, Department of Global Health, Faculty of Medicine and Health Sciences, Stellenbosch University, Cape Town, South Africa; 4 SAMRC/Wits Centre for Health Economics and Decision Science PRICELESS SA, A Division of Wits Health Consortium, University of the Witwatersrand, Johannesburg, South Africa; 5 Wits School of Public Health, Faculty of Health Sciences, University of the Witwatersrand, Johannesburg, South Africa; University of Sydney, AUSTRALIA

## Abstract

Caregivers play a critical role in promoting HIV adherence and positive mental health among adolescents living with HIV (ALHIV). However, the health and wellbeing of caregivers are often compromised by HIV-related stigma. Furthermore, in sub-Saharan Africa, the effects of HIV-related stigma are amplified by intersecting forms of stigma, such as poverty and gender. If we can identify the key drivers of HIV-related intersectional stigma, then we can develop targeted strategies to improve caregiver wellbeing. We used a mixed-method study design, utilising quantitative and qualitative baseline data from an economic incentive trial with n = 100 caregivers of ALHIV sampled from peri-urban clinics in Durban, KwaZulu-Natal, South Africa between November and December 2021. We drew on the trial’s survey dataset that examined socio-demographics, mental health and wellbeing, and stigma. We conducted descriptive statistics and fitted a linear regression model to assess correlates of HIV-related intersectional stigma using STATA (V18). In-depth interview data (n = 16 caregivers) were analysed guided by the Health Stigma and Discrimination Framework in NVivo. Of the 100 caregivers in the analysis, 86% were female, with a median age of 43 years (IQR:34–50) and 75% living with HIV. Seventy-eight caregivers reported experiencing HIV-related intersectional stigma. More than half (52%) were experiencing depressive symptoms, 51% were living in severely food-insecure households, and 59% were experiencing a high caregiver burden. Caregiver burden and total direct cost of caregiving were correlated with HIV-related intersectional stigma. Key domains from the qualitative data suggest that HIV-related intersectional stigma increased caregiver burden, diminished psychological well-being, and was linked to the cost associated with caregiving. Results highlight the need for multi-sectoral HIV-related intersectional stigma programmes that focus on economic empowerment and mental health coping strategies to equip carers with the skills needed to manage HIV-related intersectional stigma.

## Introduction

Globally, an estimated 39 million [95% CI: 33.0-45.7 million] people are living with HIV, of which 4% (1.5 million) [95% CI:1.18 million-2.19 million] are adolescents between the ages of 10 and 19 years old [1]. Sub-Saharan Africa (SSA) is currently home to approximately 85% (1.40 million) [95% CI: 1.00 million-1.85 million] of the world’s adolescents living with HIV (ALHIV) [2]. However, to date, HIV treatment outcomes among ALHIV in SSA remain sub-optimal [3]. There is a growing body of evidence that highlights the high burden of mental health issues among this population [4]. Studies have shown that adherence to and retention in ART care, as well as psycho-social outcomes of ALHIV, are highly dependent on the quality of care received from primary caregivers, who are typically mothers or other family relatives in this region [5].

As caregivers play a crucial role in the physical, emotional, and financial support of ALHIV during this complex developmental phase and treatment journey [6]. The needs and wellbeing of caregivers must be met for them to successfully fulfil their role [7]. However, studies have shown that HIV-related stigma is a barrier to performing caregiver duties optimally. HIV-related stigma usually leads to self-isolation and a lack of support and limited resources [8,9]. HIV-related stigma refers to negative attitudes, discrimination, and prejudice directed at those affected by HIV [10]. HIV-related stigma may be encountered through various mechanisms, including through peers, family, community members, and healthcare providers, often resulting in feelings of shame, guilt, depressive symptoms, and helplessness, including negative health outcomes such as non-adherence to treatment, mental health issues, and poor physical health [10–12].

According to the HIV Stigma framework, HIV-related stigma may be enacted, anticipated, or internalised [13]. Furthermore, HIV-related stigma experienced by caregivers of ALHIV may occur at multiple levels. These levels include stigma on an individual, interpersonal, and community level [14]. At the individual level, caregivers internalise stigma [10,15] by harbouring feelings of shame and guilt due to their and the adolescent in their care’s positive HIV status [16]. At the interpersonal level, caregivers often isolate themselves and their ALHIV from close networks to reduce exposure to stigma or inadvertent HIV-positive status disclosure [15]. HIV stigma at the community level is evident in SSA, where experiences of discrimination in public-sector healthcare facilities are encountered in the form of an unwelcoming environment, leading to reluctance to access ART care [17].

HIV-related stigma has been found to impact the health and wellbeing of caregivers in numerous ways. Wight et al [18] found that HIV status, race, socio-economic status, and household income were all correlated with anticipated stigma among caregivers of persons living with HIV(PLWH) in the United States [18]. Another study conducted in South Africa found that high caregiver burden and lack of social support were correlated with internalised HIV-related stigma among caregivers of PLWH [12]. Furthermore, an Ethiopian study found that caregiver HIV-related internalised stigma was linked to poor mental health outcomes, with the association higher among caregivers in rural areas than those in urban areas [19]. Caregivers of ALHIV in a Kenyan qualitative study described their experiences of enacted stigma in the form of gossip and avoidance of casual contact from community members [20]. Research also suggests that COVID-19 exacerbated experiences of HIV-related stigma, particularly for female carers, due to job losses, burden of unpaid caregiving and gender based violence [21,22]. As HIV stigma is experienced at multiple levels intersecting with other forms of stigmatised identities (e.g., gender, poverty), there is a need to further explore this complex problem using a broader framework.

The Health Stigma and Discrimination Framework is a model that describes the stigmatisation process of health conditions across various socio-ecological levels. This process includes stigma drivers, facilitators, markers (including manifestations), and outcomes. The framework draws from the HIV stigma framework but goes beyond to describe the drivers and facilitators of the various forms of stigma at each socio-ecological level. Stigma drivers, such as fear of infection, blame, and economic impacts, negatively affect the stigmatisation process. Meanwhile, facilitators like cultural norms, social support, and health policy can influence the process in both positive and negative ways, resulting in different outcomes for those affected. This framework has been used to explain HIV-related stigma as experienced by caregivers in SSA, recognising that those who care for PLWH encounter stigma, known as associative or secondary stigma, which can occur alongside other intersecting stigmas related to gender, occupation, age, and poverty [23]. According to Stangle et al [23], these intersecting stigmas occur when people are labelled with multiple stigmas referred to as stigma marking in the framework. The stigma then manifests itself in stigma experiences such as discrimination, internalised, perceived, anticipated and secondary stigma and stigma practices such as stereotypes, prejudice, stigmatising behaviour and discriminatory attitudes. These manifestations of stigma, known as stigma outcomes, then affect various outcomes for the stigmatised individual, such as access, acceptability and adherence to treatment [23].

A review of literature in SSA showed that caregivers and PLWH typically experience stigma due to low socio-economic status, poor mental health and gender [24]. These socially devalued characteristics, compounded with discrimination and prejudice faced by people affected by HIV, are referred to as HIV-related intersectional stigma (HRIS). A systematic review of systematic reviews found that HRIS was linked to negative mental health outcomes for caregivers of PLWH [25,26]. A cross-sectional pilot study conducted in Uganda found that HIV-related stigma due to caring for an ALHIV (stigma by association) was found to be significantly associated with poor mental health, particularly for female carers [27]. Furthermore, this was more pronounced among older caregivers (aged >60+) who often struggled with comorbidities [27]. A qualitative study with caregivers in KwaZulu-Natal found that medical comorbidities often heighten HIV-related intersectional stigma. Often this was because they are stigmatised due to both their HIV status and the co-morbid health conditions, particularly the co-morbidity with mental ill health [28].

Understanding the factors of HRIS among this vulnerable group of caregivers can aid in the identification of interventions to reduce the effects of stigma and enhance caregiver wellbeing. However, few studies have examined the factors of HIV-related intersectional stigma in the context of caregivers of ALHIV and COVID-19 in SSA. Even though extensive research has examined the experiences and needs of ALHIV, we know very little about the perspectives of their caregivers, who are central to supporting disclosure, HIV treatment adherence, and their psychosocial wellbeing. The current gap in our understanding of how household- and caregiver-level correlates shape adolescent outcomes, particularly within the context of caregiver stress, stigma and coping, is needed. Therefore, this study aims to contribute to the body of knowledge on HIV-related intersectional stigma by (i) identifying the correlates of HIV-related intersectional stigma; and (ii) understanding the lived experiences of HIV-related intersectional stigma among caregivers of ALHIV in KwaZulu-Natal, South Africa, during the COVID-19 pandemic.

## Methods

### Ethics statement

Written informed consent was obtained from all participants. Ethics approval was obtained from the South African Medical Research Council’s Human Research Ethics Committee (EC036–8/2021), and permission to conduct this research within the healthcare facilities was obtained from the South African National Department of Health (KZ_202112_005). Participants received cash (R50, ~ 3 USD) per interview completed to reimburse them for their time. As baseline interviews were conducted face-to-face, participants also received a lunch pack and transport money to the value of R100 (~ 6.10 USD). The trial has been registered in the Pan African Clinical Trials Registry (PACTR) PACTR202203585402090; URL: https://pactr.samrc.ac.za/.

### Study design and participants

A mixed method secondary analysis study design was employed, drawing on baseline quantitative and qualitative data collected from the pilot Caregiver Wellbeing (CWeL) trial (PACT no: PACTR202203585402090, https://pactr.samrc.ac.za/) [29]. The trial evaluated an economic incentive package for improving caregiver wellbeing and was conducted between November 2021 and March 2022 in the eThekwini Metropolitan area of KwaZulu-Natal, South Africa. Participants were primary caregivers above 18 years of age taking care of ALHIV aged between 10–19 years who were accessing care in HIV clinics located in peri-urban and urban settings, regardless of gender. There was a total of 100 caregivers who were randomised 1:1 to intervention or control arms. The sample size was determined in the pilot trial by considering the primary outcome, where a minimum sample size of 15 per arm would be required for 80% power to detect a treatment effect. Therefore, a sample size of 100 was determined, assuming n = 20 refused to participate or were lost to follow-up. This study was approved by the South African Medical Research Council (SAMRC) Health Research Committee. Its ethical approval reference number is: EC006-4/2022.

### Data collection

Both quantitative and qualitative data in the trial were collected by trained research fieldworkers through face-to-face interviews. The interviews were conducted in the participants’ preferred language. The baseline survey was interviewer-administered and was captured electronically on REDCap (Version 7.4.1) using electronic tablets. The survey collected data on demographics, socio-economic status, mental health and wellbeing. The baseline in-depth interviews were based on topic guides that explored the cost of caregiving, social and financial support, mental and physical health, internalised or experienced stigma, and how the COVID-19 pandemic shaped their experiences. The authors accessed the anonymised data on the 10^th^ of August 2022; the authors did not have access to information that could identify the participants.

### Measurements

#### Quantitative data.

Data were gathered using the following measurements, which were translated into isiZulu and back-translated into English to ensure accuracy, with cognitive understanding of key outcome measures assessed during a brief pilot phase. The selection of measures was informed by expert guidance from an NIH-funded workshop attended by the principal investigator, as well as by a systematic review that outlined key conceptual frameworks and recommended measures for assessing HIV-related internalised stigma [30]. These tools were piloted for comprehension and back-translated without any adaptations.

***Demographics, labour and employment, and household income:*** This section was informed by the National Income Dynamics Study (NIDS) – Coronavirus Rapid Mobile CRAM survey (i.e., NIDS-CRAM survey) [18]. The NIDS-CRAM survey assessed the impact that the COVID-19 lockdown had on caregivers and their households. This tool informed the household income questions and guided questions around caregiver background (demographics, dwelling type, household occupants, household dwellers’ ages), labour and income (employment, employment type, remuneration frequency, income range), and household and social outcomes (number of children on support grants, number of elderly on pension grants, other forms of government support, source(s) of income, and the main source of income).
***HIV-related intersectional stigma:*** The Everyday Discrimination Scale [13] was used to measure HIV-related intersectional stigma. The scale contains 10 items with scores ranging from 1 to 5, with 1 = “Almost every day” and 5 = “Less than once a year or never”. A lower score indicates higher stigma and discrimination. An example of one of the items includes: “You are treated with less courtesy or respect than other people are.”
***Mental health (Depressive symptoms):*** The CES-D-10 was used to identify symptoms of depression. It comprises 10 items that assess how an individual felt in the past week. Eight items cover negative feelings, such as “I was bothered by things that usually don’t bother me”, and the remaining two items, are stated in the positive (e.g., “I was happy”) [14]. Participants responded to the items on a four-point Likert scale, where 1 = “Rarely or none of the time (1 day)”, 2 = “Some or a little of the time (1-2 days)”, 3 = “Occasionally or a moderate amount of time (3-4 days)” and 4 = “Most or all of the time (5-7 days)”. The CES-D-10 has been psychometrically evaluated in a South African sample and has shown good validity and reliability [31]. A cut-off point of 12 is deemed optimal to correctly classify individuals with a diagnosis of depression for a South African sample [15].
***Caregiver burden and happiness:*** The CarerQol-7D scale was used to measure caregiving burden (https://www.imta.nl/carerqol/) is made up of two parts: (i) the CarerQol-7D, which describes the care situation in terms of the negative and positive effects of caregiving (e.g., “I have no/some/a lot of financial problems because of my care tasks”) on a 3-point Likert scale where 1 = No, 2 = Some and 3 = A lot of, and (ii) the VAS which measures happiness on a scale from 0 to 10 (0 = completely unhappy and 10 = completely happy).
***Psychological wellbeing:*** The Mental Health Continuum short form was used to measure psychological wellbeing. This scale is a short 14-item scale, with 3 items examining subjective wellbeing, 6 items examining psychological wellbeing, and 5 items that measure dimensions of Keyes [32] social wellbeing model, particularly social integration and social contribution (e.g., “During the past month, how often did you feel that you belonged to a community like a social group or a neighbourhood?”. It uses a six-point Likert scale, ranging from 1 = never to 6 = every day. The scale has been validated with Setswana-speaking females in the North West province of South Africa with acceptable internal consistency (α = .74) [16].
***Household food insecurity:*** The Food Insecurity Experience Scale was used to assess household and individual food security, which has demonstrated good internal validity in SSA (α = .78) [17]. It is an 8-item scale that probes respondents’ access to adequate food (e.g., “During the last 12 months, was there a time when, because of lack of money or other resources, you were worried you would not have enough food to eat?”). The scale measured food insecurity with scores ranging from 1 = Yes, 2 = No, 3 = Don’t know, 4 = Refused.

#### Qualitative data.

Qualitative data were collected from 8 caregivers in each arm of the trial at baseline. These participants were purposively sampled for a semi-structured in-depth face-to-face interview after the quantitative interview. Participants were purposively selected to ensure that the sample was reflective of the following factors at baseline (i.e., age (< 60 vs. ≥ 60 years of age), households above and below the lower bound poverty line/threshold of R840 per capita [33], psychological wellbeing score, and HIV status).

### Analysis

For quantitative data, continuous variables were expressed as medians (IQR) and categorical variables were expressed as frequencies (proportions). Simple linear regression was used to assess the relationship between the HIV-related intersectional stigma score and covariates (namely, depression, mental health, caregiver burden, and demographic factors). Multivariable linear regression was used to assess the predictors of intersectional stigma. A sensitivity analysis using robust Poisson regression was done to assess the predictors of intersectional stigma. All variables with a p-value <0.15 in the univariable model were included in the multivariable model, and age was used as an a priori variable. All statistical analyses were performed using Stata (Stata Corp, College Station, TX, V 18). A complete case analysis was done since all of the variables had no missing data, thus no imputation method was carried out. All statistical analyses were considered significant at 5% level.

For the qualitative data, we used a framework method for analysis [19] guided by the Health Stigma and Discrimination Framework, which was done in 7 stages. The Framework was selected based on recommendations from a systematic review [30] of studies examining internalised stigma, as well as guidance from the first author, who has been leading work in HIV stigma. For Stage 1 (Transcription), all electronic audio files were transcribed and checked by a member of the team. Stage 2 (Familiarisation) entailed listening to the audios and reading the transcripts and field notes. Stage 3 (Coding) entailed dual coding of a few transcripts using NVivo 12 [19]. Thereafter, in Stage 4, the Health Stigma and Discrimination Framework was used to code data from the initial transcripts, which were grouped into categories related to race, gender, sexual orientation, class, and occupation. Once the framework was finalised, this was applied to the remaining transcripts using NVivo, and the file was continually updated with any new codes and categories arising from the data (Stage 5: Applying the analytical framework). In Stage 6 (Charting data into a framework matrix), key quotes were extracted from transcripts that exemplify a category, and a matrix (i.e., a summary of categories per transcript) was produced using NVivo. In the final stage, Stage 7 (Interpretation), we conducted with both the field and investigator teams, where differences and similarities identified in the data (e.g., perceptions by age, household socio-economic status) were discussed. The introduction of multiple perspectives in the interpretation process reduces potential bias and supports the triangulation of interpretations, ensuring that results are based on the diversity of the data rather than researcher bias.

## Results

### Quantitative results

Of the 100 caregivers in the CWeL trial, 86 were female with a median age of 43 years [IQR:34–50] while over half (69%) had some form of secondary education and 75% were living with HIV (Table 1). At the time of the study, 59% of the participants were unemployed, and 88% of the participants were receiving social grants from the government. Slightly above half (52%) of caregivers reported experiencing depressive symptomology. Half of the caregivers experienced either moderate or languishing levels of wellbeing. Most of the caregivers lived in formal houses (85%), more than half of them (69%) reported experiencing moderate to severe food insecurity, and 45% were living below the upper-bound poverty line set at ZAR 1417,00 (~70,38 USD) as at 2022 [33].

**Table 1:** Description of caregivers of ALHIV in the sample (N = 100).

	Characteristic	n (%)
**Socio-demographics**		
	Median age [IQR⁵]	43[34 - 50]
	Age range (years)	
	20-29	13
	30-39	30
	40-49	32
	50-59	16
	60+	9
	Sex	
	Female	86
	Male	14
	Highest grade completed	
	Primary	29
	Secondary	71
	Employment status	
	Unemployed	59
	Employed	41
**Health status**		
	HIV status (self-reported)	
	HIV positive	89
	On ART⁷	85
		
**Mental health and wellbeing**	Depressive symptoms (CESD-10^1^)	
	Yes	52
	No	48
	Psychological wellbeing (MHC-SF¹)	
	Flourishing	49
	Moderate to languishing	51
	Caregiver burden (CarerQoL-7D³)	
	High	59
	Low	41
	Caregiver happiness (CarerQoL-VAS)	
	High	31
	Low	69
	Ever experienced HIV-related intersectional stigma	
	Yes	78
	No	22
**Household characteristics**	Type of household dwelling	
	Formal	85
	Informal	15
	Total direct cost of caregiving^7^	
	<=R100	39
	>R100	61
	Household Food insecurity^2^	
	Mild	31
	Moderate	18
	Severe	51
	Social Grant Recipient	
	Yes	88
	No	12
	Household poverty threshold⁴	
	Below poverty line threshold	49
	Above poverty line threshold	51

¹**CESD-10:** Centre for Epidemiological Depression Scale.

²**MHC-SF**: Mental Health Continuum Short Form.

³**CarerQoL:** Carer Quality of Life.

⁴**Poverty threshold:** Upper bound poverty threshold set at R1417 (77.30 USD) as of 2022.

⁵**IQR:** Interquartile range**`.**

**^6^ART: Antiretroviral therapy.**

**^7^ Total direct cost of caregiving:** out of pocket payments associated with taking of an ALHIV.

^8^**FIES**: Food Insecurity Experience Scale.

Table 2 presents a comparison of HIV-related intersectional stigma with identified correlates included in the study, based on a review of the literature. Males (57.14%) were more likely to experience HIV-related intersectional stigma than females (51.16%), with caregivers between the ages of 40–49 (62.50%) being the most likely to have experienced HIV-related intersectional stigma and least likely in caregivers 60 years and above (33.33%). HIV-related intersectional stigma was more prevalent among caregivers living with HIV (52.81%) than those living without HIV (45.45%). Caregiver burden was more prevalent among participants experiencing higher burden of caregiving (61.02%) than those who were experiencing lower levels of caregiver burden (39.02%). Participants experiencing severe food insecurity (82.35%) were also experiencing higher levels of HIV-related intersectional stigma compared to those with mild (64.71%) and moderate (55.56%) experiences of food insecurity.

**Table 2:** Comparison of ever experienced HIV-related intersectional stigma scores by selected variables N = 100.

Variable	Ever experienced HIV-related intersectional stigma n (%)		
	No (n = 48)	Yes (n = 52)	p-value
**Sex**			0.680
Male	6 (42.86)	8 (57.14)	
Female	42 (48.84)	44 (51.16)	
**Age range**			0.360
20-29	8 (61.54)	5 (38.46)	
30-39	13 (43.33)	17 (56.67)	
40-49	12 (37.50)	20 (62.50)	
50-59	9 (56.25)	7 (43.75)	
60+	6 (66.67)	3 (33.33)	
**HIV status**			0.650
HIV-	6 (54.55)	5 (45.45)	
HIV+	42 (47.19)	47 (52.81)	
**Employment**			0.490
Unemployed	30 (50.85)	29 (49.15)	
Employed	18 (43.90)	23 (56.10)	
**Highest grade completed**			0.170
Primary	17 (58.62)	12 (41.38)	
Secondary	31 (43.66)	40 (56.34)	
**Depressive symptoms (CESD-10)**			0.079
No	22 (59.46)	15 (40.54)	
Yes	26 (41.27)	37 (58.73)	
**Psychological wellbeing (MHC-SF)**			0.230
Flourishing	12 (60.00)	8 (40.00)	
Moderate to languishing	36 (45.00)	44 (55.00)	
**Household food insecurity**			0.007
Mild	22 (70.97)	9 (29.03)	
Moderate	8 (44.44)	10 (55.56)	
Severe	18 (35.29)	33 (64.71)	
**Caregiver burden**			0.030
Low	25 (60.98)	16 (39.02)	
High	23 (38.98)	36 (61.02)	
**Total direct cost of caregiving**			0.091
Low	48 (49.48)	49 (50.52)	
High	0 (0.00)	3 (100.00)	

Table 3 shows results from the linear regression model for the correlates of HIV-related intersectional stigma among caregivers of ALHIV. Univariable results show a correlation between high total direct cost of living and HIV-related intersectional stigma (β = 12.79, CI = 2.19 to 23.38, p = 0.019). Severe household food insecurity was associated with HIV-related intersectional stigma (β = 6.09, CI = 2.02 to 10.17, p = 0.004). Caregiver burden was also associated with HIV-related intersectional stigma (β = 6.06, CI = 2.48 to 9.64, p = 0.001). In the multivariable model, severe household food insecurity (β = 4.26 (-0.70 to 9.22), CI = -0.70 to 9.22, p = 0.091), and depressive symptoms (β = 1.35 (-5.58 to 8.81), CI = -5.58 to 8.81, p = 0.529) did not demonstrate statistical significant associations with HIV-related intersectional stigma. Caregiver burden (β = 4.50 (0.19 to 8.81), CI = 0.19 to 8.81, p = 0.041) was associated with HIV-related intersectional stigma. A sensitivity analysis was done using the HIV-related intersectional stigma as a binary variable. High total direct cost of living was associated with HIV-related stigma (RR = 1.67, CI = 1.13 to 2.47, p = 0.011). Caregivers who had a high total direct cost of living were 1.67 times more likely to be at risk of HIV-related intersectional stigma compared to those with low cost of living, Table 4.

**Table 3:** Univariable and multivariable linear regression model coefficients for correlates of HIV-related intersectional stigma among caregivers of ALHIV (N = 100).

	Univariable		Multivariable	
Characteristic	β (95% CI⁹)	p-values	β (95% CI)	p-values
**Caregiver age range (years)**				
**30-39**	0.19 (-6.01 to 6.40)	0.950	-1.04 (-7.10 to 5.02)	0.734
**40-49**	1.99 (-4.15 to 8.14)	0.521	0.10 (-5.95 to 6.14)	0.975
**50-59**	1.71 (-5.27 to 8.69)	0.627	-1.42 (-8.27 to 5.43)	0.681
**60+**	-2.76 (-10.87 to 5.34)	0.501	-4.86 (-12.8 to 3.08)	0.227
**Type of dwelling**				
**Formal house**				
**Informal house**	1.50 (-3.70 to 6.69)	0.569		
**Education**				
**Primary school**				
**Secondary school**	1.14 (-2.95 to 5.24)	0.581		
**Employment**				
**Employed**				
**Unemployed**	-2.16 (-5.92 to 1.59)	0.256		
				
**Total direct cost of caregiving**				
**Low**				
**High**	12.79 (2.19 to 23.38)	0.019	9.95 (-0.67 to 20.56)	0.066
**Household food insecurity (FIES⁶)**				
**Mild**				
**Moderate**	2.46 (-2.84 to 7.75)	0.360	1.58 (-4.04 to 7.19)	0.579
**Severe**	6.09 (2.02 to 10.17)	0.004	4.26 (-0.70 to 9.22)	0.091
**Depressed (CES-D 10)**				
**Yes**	2.64 (-1.17 to 6.46)	0.172	-1.35 (-5.58 to 8.81)	0.529
**No**				
**Psychological wellbeing**				
**Flourishing**				
**Moderate to Languishing**	2.33 (-2.30 to 6.95)	0.321		
**Caregiver burden (CarerQoL-7D)**				
**High**	6.06 (2.48 to 9.64)	0.001	4.50 (0.19 to 8.81)	0.041
**Low**				
**Caregiver happiness**				
**High**	-2.71 (-6.51 to 1.08)	0.159		
**Low**				

*only variables with p < 0.15 are included in the multivariable model except for age which was selected as an apriori variable.

⁶Food Insecurity Experience Scale.

**Table 4:** Univariable and multivariable robust Poisson regression model for HIV-related intersectional stigma among caregivers of ALHIV (N = 100).

	Univariable		Multivariable	
Characteristic	RR (95% CI)	p-values	RR (95% CI)	p-values
**Caregiver age range (years)**				
**30-39**	1.47 (0.69 to 3.14)	0.317)	1.27 (0.61 to 2.64)	0.518
**40-49**	1.63 (0.77 to 3.41)	0.200	1.30 (0.64 to 2.67)	0.469
**50-59**	1.14 (0.47 to 2.77)	0.776	0.88 (0.37 to 2.07)	0.767
**60+**	0.87 (0.27 to 2.76)	0.809	0.67 (0.22 to 2.06)	0.480
**Type of dwelling**				
**Formal house**				
**Informal house**	1.19 (0.74 to 1.89))	0.473		
**Education**				
**Primary school**				
**Secondary school**	1.36 (0.84 to 2.20)	0.209		
**Employment**				
**Employed**				
**Unemployed**	1.14 (0.78 to 1.66)	0.492		
**Household food insecurity (FIES⁶)**				
**Mild**				
**Moderate**	1.91 (0.96 to 3.82)	0.066	1.74 (0.83 to 3.64)	0.140
**Severe**	2.23 (1.24 to 4.02)	0.008	1.90 (0.93 to 3.87)	0.076
**Depressed (CES-D 10)**				
**Yes**	**1.45 (0.93 to 2.26)**	0.102	1.04 (0.66 to 1.67)	0.854
**No**				
**Psychological wellbeing**				
**Flourishing**				
**Moderate to Languishing**	1.38 (0.77 to 2.44)	0.278		
**Caregiver burden (CarerQoL-7D)**				
**High**	1.56 (1.01 to 2.42)	0.044	1.21 (0.78 to 1.92)	0.410
**Low**				
**Total direct cost of caregiving**				
**Low**				
**High**	**1.98 (1.62 to 2.41)**	<0.001	1.67 (1.13 to 2.47)	0.011
*only variables with p < 0.15 are included in the multivariable model				
⁶Food Insecurity Experience Scale				

### Qualitative findings

The first domain, according to the Health Stigma and Discrimination Framework model, outlines the *drivers and facilitators of HIV-related intersectional stigma*. The drivers perpetuating HIV-related intersectional stigma, according to participants, include not sharing information about their HIV-positive status or the use of ART. Being hypervigilant about not sharing information about the HIV status of the ALHIV and the use of ART, is linked to the increased burden of caregiving. The increased burden of caregiving was also found in the quantitative data to be a significant contributor to the experiences of HIV-related intersectional stigma. This is described by two participants as follows:

“They [the children] know except the ARVs one … I do not discuss that one [referring to taking ART]... Yes, there is a lot of discrimination: as you can see her being so sick, she takes that, the ARVs. That is what I dread. Or if they have seen someone sick and has lost weight: oh, well, this one was found out that she has that thing. Yes, now I saw that, no, I will just survive … Yes, the gossip too much” (Participant 3, Female, Age 46)

“There is an elderly girl that she [referring to the child in her care] goes and visits. That I am crossing my finger that she must never [know – knowing about the parents HIV positive status] because you know what it is like with the children.” (Participant 8, Female, Age 66)

In both the examples above, the drivers toward intersectional stigma are fuelled by not sharing the HIV status of the ALHIV with others in the community, as outlined by Participant 8. This hypervigilance of being aware of not sharing the HIV status with others adds to the caregiver burden experiences, which is linked to the increased fears of anticipated intersectional stigma. There is also evidence of facilitators, which are those factors that either positively or negatively influence the intersectional stigma, as discussed by Participant 4, who outlines some of the assumptions that negatively contribute to the experiences of HIV-related intersectional stigma:

“No, I did not start with the treatment … I just was not ready; I was afraid that maybe I will take them and then default.” (Participant 4, Female, Age 31)

“They have this assumption that if you have HIV, you have been living on the fast lane.You find that others…like you will find that at the time people were taking Navirapine. I would have taken it but it seemed my blood was able not to show. Sometimes they would speak anyhow and say: those parents who do not consider the children. People are not the same. Some do understand, you see, people are not the same.” (Participant4, Female, Age 31)

When Participant 4 reflects on defaulting treatment and the experience of how others treat her. These facilitators of intersectional stigma are based on the community norms and how these perpetuate stigma and feelings of depression. Findings from the quantitative results, suggest that the association between caregiver burden and HIV-related intersectional stigma, even though depressive symptoms were not significantly associated.

The second domain is *stigma marking,* which is a result of the drivers and facilitators related to HIV, and how people or groups of people are perceived based on their health condition. This is evidenced when stigmatised people or groups of people are marked with multiple stigmas, as the participants discussed below, the first is an example of where the caregiver outlines the hurt experienced due to the child’s HIV status and the second example is where a caregiver does not want to share the status with certain individuals due to an increase in multiple forms of stigma:

“See, my heart is in pain because I have not heard of any child in the community that has AIDS. I sometimes wonder how it feels to her [referring to the child in his care who is HIV positive] because the Social Workers tell her directly what the cause of her sickness [referring to HIV] is. I just wonder how she would feel because as she is taking medication, all she knows is that she is sick. What the cause of her sickness is, she does not know, you see. So, I my heart I say how painful the growth of this child is because she is still too young. One day she will grow and know that she has this sickness.”(Participant 2, Male, Age 57)

“I do not have a problem that I am HIV [positive] but there are people that you do not want for them to know.” (Participant 4, Female, Age 31)

The two excerpts above detail the caregiver burden, more specifically, the psychological toll on the caregiver, which is experienced as a result of the HIV status of the ALHIV in their care. The HIV status of the ALHIV in their care is the marker that contributes to the intersectional stigma experienced. The qualitative domain integrates with the quantitative findings of how the levels of caregiver burden as a result of HIV status significantly predicts the levels of intersectional stigma experienced by the caregivers of ALHIV. The stigma experienced as part of the *stigma marking* is further perpetuated in the third domain of *experiences and practices.* The experiences are the lived realities of the caregivers and their adolescents, which can be classified as stigmatising behaviour. Two participants unpack their experiences of how persons in the community further heighten the stigmatising experiences through their behaviours that contribute to the increased burden of caregiving and HIV-related intersectional stigma:

“They do [referring to being stigmatised by persons in the community], you see, because if you are having something, you do not share it through your mouths. If they know that you have something [referring to HIV], they give you a separate glass because you no longer the same as them.” (Participant 2, Male, Age 57)

“Yes, they [referring to persons in the community] very scared of me … I really think it’s because of my HIV status … No, but they always ask what treatment I always fetch from the clinic, and I tell them its my medication for psychosis treatment. And they tell me I’m lying its HIV treatment medication, because if it was psychosis treatment, I would be [on a] social grant.” (Participant 12, Male, Age 23)

In addition to the experiences, the practices, which are the beliefs, attitudes and actions, that perpetuate the HIV-related intersectional stigma as described by Participant 2:

“Some make a joke of it [referring to having HIV] saying they are not ashamed because to them when they collect the medication it is as if they are buying something from the shops. If they see you carrying the treatment, they will say it is this and that. So, I am a bit nervous because as an elderly person, what would people say; what sort of an elderly man am I to have this kind of thing [referring to having HIV]. Nobody else knows about mine except my family.” (Participant 2, Male, Age 57)

The stigma experiences and practices shared above are further examples of the age of caregivers (see Participant 2’s reflection) and how it contributes to the ‘psychological’ burden of caregiving, such as feeling worthless and guilty about their own HIV status, which are linked to the stigma experienced. These experiences and practices are the ‘manifestations’ that lead to the fourth domain, which is *outcomes* that result in health and social impacts experienced due to the intersectional stigma. One of the outcomes highlighted by one of the participants was related to lying to the child living with HIV about his diagnosis and the medication to be taken as a result of the stigmatising behaviours:

“He [referring to the child in her care that is HIV positive] would ask me: mother when will these pill finish? It means this is for the rest of my life. Sometimes you just have to say no they will stop you from taking them. Sometimes I say no, they will stop you. Just take them for at least five years and on the sixth year they will make you stop, you see that. Yes, things like that. It is difficult I would not lie. It is difficult my sister.”(Participant 5, Female, Age 43)

“Not having enough money means you cannot run away you have to stay in that area even if people are not treating you well because you can’t go anywhere” (Participant 6,Female, 39).

Integrating the quantitative findings with the qualitative findings suggests that the correlates of caregiver burden, which is psychological, and the high total direct cost of living, which is financial, are significant predictors of HIV-related intersectional stigma, and how the domains from the qualitative data evidence these correlates as predictors of intersectional stigma based on the experiences of the caregivers of ALHIV.

## Discussion

The study used the Health Stigma and Discrimination Framework to understand the factors associated with and the lived experiences of HIV-related intersectional stigma among caregivers of ALHIV. The results indicate that caregiver burden, and high total direct cost of caregiving are correlated with HIV-related intersectional stigma. Caregivers of ALHIV expressed the need to conceal their HIV status and that of the ALHIV due to fear or anticipation of stigma. Caregivers felt pressured by gender norms in their community. These caregivers feared they would be seen as promiscuous if their HIV status were known. Furthermore, male caregivers were concerned about how taking ARVs might affect their social standing. Financial constraints were identified as a barrier to escaping stigma in their communities, which could be linked to the total direct cost of caregiving. Caregivers, however, felt stuck in the communities where they were already stigmatised, or their HIV status had become public knowledge. This evidence aligns with the framework’s identified intersecting stigmas related to gender, occupation, and poverty, all of which often intersect with health-related stigmas [23].

Our quantitative results found that HIV-related intersectional stigma was associated with caregiver burden, which could be psychological in nature. Our results reflect similar findings from a global systematic review which suggest that increased caregiver burden, depression, stress and distress were all associated with higher stigma scores [34]. In addition, caregivers with a high direct cost of caregiving were more likely to experience HIV-related stigma, but this was not significant when compared to those with a lower average direct cost of caregiving. This highlights the need for interventions that will economically empower caregivers to alleviate the economic burden associated with caregiving and the stigma that accompanies it [35].

According to the Health Stigma and Discrimination Framework, stereotypes and prejudices are identified as drivers of HIV-related intersectional stigma. This was evident in caregivers’ lived experiences in the qualitative interviews, where some caregivers expressed the need to isolate their ALHIV and themselves in their communities due to fear of stigma. The social isolation because of internalised stigma, as described by the lack of support and community ridicule, may lead to feelings of helplessness and low self-esteem, which might contribute to decreased wellbeing and increased caregiving burden. The high caregiver burden associated with higher stigma was also evident in the caregivers’ experiences. They had to keep finding new ways to explain to the ALHIV why they are taking ARVs or keep them in treatment. This is due to HIV non-disclosure and the constant need to hide their HIV status and the status of ALHIV to shield them from stigma from the community and family members [5]. Treatment fatigue from the ALHIV also strains the caregiver-ALHIV relationship. This strain could further increase the caregiver burden, as the caregiver constantly tries to convince the ALHIV to stay in ART care [36].

It is clear from the results that caregivers of ALHIV are prone to stigmatisation due to possessing various stigmatised identities. These identities include their HIV status, socio-economic status, mental health challenges and gender norms. Males in our sample were more likely to experience HIV-related intersectional stigma compared to females, although our study included a small number of males. An Ethiopian cross-sectional study with primary caregivers of children and adolescents with mental illness, however, found that being a mother (Adjusted Odds Ratio (AOR) = 2.8, 95% CI: 1.59, 4.91), poor social support (AOR = 3.9, 95% CI: 1.59, 6.13), and symptoms of depression (AOR = 2.9, 95% CI: 1.88, 3.65) were factors significantly associated with perceived stigma [37]. A similar finding in our qualitative data was that one caregiver expressed that people assume that, as a woman living with HIV, it means that you have been promiscuous in the past. Such gender norms facilitate HIV-related intersectional stigma as described in the framework, but also contribute to the psychological toll of the burden of being a caregiver.

The Health Stigma and Discrimination Framework recognises the social aspect of HIV-related intersectional stigma, especially in the context of caregivers of ALHIV in SSA. Caregivers of ALHIV in SSA have various socially devalued characteristics. The framework also poses a challenge for interventions targeting HIV-related intersectional stigma as it describes this phenomenon as a socio-ecological problem that calls for multi-level interventions [38]. Our findings suggest that the burden of caregiving, which could be psychological, and the total direct cost of caregiving, which is financial, need to be considered in multi-level interventions to address HIV-related intersectional stigma. Although the Health Stigma and Discrimination Framework is applicable across various health conditions. It is not explicit in cases of co-morbidities and how these intersect with devalued characteristics. This is particularly true within our sample of caregivers, where a majority of them are living with HIV and likely to be experiencing mental health problems. Moreover, although we found a lot of literature focusing on the adaptation of the Health Stigma and Discrimination Framework for HIV stigma [39–41], there was a lack of studies focusing on sub-populations such as caregivers of ALHIV.

Our results provide the following guidance for programmes and policies: There is a need for gender-transformative economic interventions targeting caregivers of ALHIV. These interventions should aim to promote gender equity within relationships, enhancing communication skills, and improving wellbeing. These could include positive mental health coping strategies, preventing intimate partner violence, sexual health, goal setting, spending and saving, as well as applying and keeping jobs [42]. There was some evidence of food insecurity in our sample, with some evidence suggesting that caregivers from food-insecure households reported higher HIV-related intersectional stigma [43]. Cash transfers have been shown to be of benefit in addressing high direct cost of caregiving, which could result in food insecurity and increased poverty, particularly in South Africa and as seen in the current study [44,45]. There is a need for school-based intervention targeting HIV-related intersectional stigma among adolescents [44]. A Ugandan study found that support at school, either from teachers or classmates, was associated with HIV disclosure and lower HIV-related stigma [45]. Social alienation and isolation were a recurring theme in the qualitative interviews. Caregivers can benefit from social support groups or spaces where caregivers can interact with one another so they feel less alone, easing the psychological burden of caregiving [46]. In addition, some of the policy recommendations based on the findings could include: (i) implementation of community-wide campaigns to address stigma and promote accurate knowledge about HIV and treatment, (ii) expanding current social development policies and programmes, such as cash incentives, child grants or caregiver stipends, to alleviate high caregiver burden and financial strain on households affected by HIV related intersectional stigma, (iii) considering the inclusion of routine mental health screening as part of the referral pathway for caregivers and ALHIV in HIV care frameworks, (iv) fostering anti-discrimination policies to minimise stress due to external stigma among caregivers and ALHIV. There are also recommendations for programmes to address correlates related to HIV-related intersectional stigma: (i) such as community-based peer education initiatives to reduce stigma and foster more inclusive and affirming environments, as well as (ii) establishing support groups to provide psychosocial support and minimise caregiver stress, while also fostering resilience and stress management, in addition to the mental health support provided as part of a referral pathway framework.

Our study has the following strengths: we adapted the Health Stigma and Discrimination Framework to guide our understanding of the correlates and experiences of HIV-related intersectional stigma. Participants were randomly selected to participate in the study and both males and females were recruited. Moreover, we collected both quantitative and qualitative data to measure both the correlates and experiences of HIV-related intersectional stigma. However, the study’s results should be considered, noting the following limitations: (i) cross-sectional studies only provide a snapshot and not temporal trends to assess causality; (ii) our sample included only 100 caregivers, and the sample size may be increased to increase statistical power. (iii) Another limitation to consider is the collection of self-reported data, which can also be linked with the potential social desirability bias in the reporting. (iv) In addition to the possibility of selection bias, the trial only recruited caregivers of ALHIV accessing clinics. It is likely that carers and ALHIV not in care may be exposed to higher levels of HIV-related intersectional stigma. (v) The stigma scale was not psychometrically validated in South Africa, along with no local study categorising total stigma scores. (vi) Furthermore, when reflecting on the use of the framework, the study did not collect data on the broader structural (e.g.: health policies and institutional discrimination) and community-level factors (e.g.: socio-cultural norms and collective behaviours) [30].

## Conclusion

The study aimed to measure the correlates of HIV-related intersectional stigma and understand the experiences of caregivers of ALHIV during COVID-19. We found that HIV-related intersectional stigma faced by caregivers of ALHIV in KwaZulu-Natal is a pressing issue associated with higher caregiver burden and increased direct cost of caregiving. To address this challenge effectively and ensure the well-being of both caregivers and the ALHIV, several policy recommendations are essential, including caregiver-centred interventions, women’s empowerment and education, ongoing research and data collection, and multisectoral collaborations. Even though these recommendations offer valuable alternatives, it is essential to assess their feasibility and scalability in light of contextual factors, recognising that communities and health systems may differ significantly in resource availability, burden of disease, access and demand, as well as prevailing values and beliefs.

## Supporting information

S1 Table Description of variables. (DOCX)
