# Supplementary material for: The correlates and experiences of HIV-related intersectional stigma among caregivers of adolescents living with HIV during COVID-19 in KwaZulu-Natal, South Africa: Results from a mixed method study
Source: PLOS Glob Public Health. 2026 Apr 29;6(4):e0004296. doi: 10.1371/journal.pgph.0004296 (PMC13127923; doi:10.1371/journal.pgph.0004296)
Supplement: S1 Table — (DOCX) [file pgph.0004296.s001.docx]

# S1 Table: Description of variables

| **Variables** | **Survey questions** | **Survey response options** | **Grouping and recoding of survey response options** |  |
| --- | --- | --- | --- | --- |
| Caregiver age range | How old are you? | Number (Min= 18, Max= 95) | 1 = > 20  2 = 20-29  3 = 30-39  4 = 40-49  5 = 50-59  6 = 60+ |  |
| Sex | What is your sex? | 1 = Male 2 = Female 99 = Refused to answer | 1 = Male 2 = Female |  |
|  |  |  |  |  |
| HIV status | Do you currently have any of the following conditions? | 1 = TB  2 = Asthma  3 = Diabetes  4 = Hypertension  5 = HIV  6 = Cancer  7 = COVID-19  8 = Arthritis  9 = kidney disease  10 = Heart condition  11 = HIV – On Art  12 = HIV – Not on ART  13 = Mental health-related e.g., anxiety, depression, bipolar)  14 = Other  99 = Refused  14 = No | 1 = HIV positive [if 5, 11 or 12]  0 = HIV negative [if none of 5, 11 or 12] |  |
| Type of dwelling | What type of dwelling or house do you live in? | 1 = Formal (house/flat made of brick and cement) 2 = Informal house (e.g., made of wood or tin)  3 = Traditional house (e.g., mud house) 99 = Don’t know | 1= Formal  2 and 3 = Informal |  |
|  |  |  |  |  |
| Highest school grade completed* | What is the highest standard of schooling that you have completed or passed | 0 = Not completed any grade 1 = grade 1 2 = grade 2 3 = grade 3 4 = grade 4 5 = grade 5 6 = grade 6 7 = grade 7 8 = grade 8 9 = grade 9 10 = grade 10 11 = grade 11  12 = grade 12 99 = Prefer not to answer | 1-8 = [< grade 9]  9-12 = [≥ grade 9] |  |
| Employment status | What do you do to make money? | 1 = Employed 2 = Self-employed 3 = Temp/casual worker 4 = Do odd jobs 5 = Unemployed | 1 = Employed [if Yes to any 1-4]  0 = Unemployed [if Yes to 5] |  |
| Total direct cost of caregiving | In the past month, how much have you spent on: | 1 = Transport taking your child to and from health facilities? (e.g., clinic, hospital, general practitioner etc.)  2 = Admission and consultation fees? 3 = Medication 4 = Food while at the health facility 5 = Any fees paid for diagnostic procedures  6 = Cost to somebody else accompanying your child to healthcare facility other than yourself?  7 = Cost to somebody else taking care of your child when you not around (e.g., at work)? | Sum of 1-7 |  |
| Food Insecurity Experience Scale: Household referenced | During the last 12 months, was there a time when you or others in your household were worried about not having enough food to eat because of a lack of money or other resources? | 0 = Never 1 = Sometimes 2 = Often 99 = Prefer not to answer | 1 = Mild  2 = Moderate  3 = Severe |  |
|  | During the last 12 months, was there a time when you or others in your household were unable to eat healthy and nutritious food because of a lack of money or other resources? | 0 = Never 1 = Sometimes 2 = Often 99 = Refused to answer | 1 = Mild  2 = Moderate  3 = Severe |  |
|  | During the last 12 months, was there a time when you or others in your household ate only a few kinds of food because of lack of money or other resources? | 0 = Never 1 = Sometimes 2 = Often 99 = Refused to answer | 1 = Mild  2 = Moderate  3 = Severe |  |
|  | During the last 12 months, was there a time when you or others in your household ate less than you thought you should because of a lack of money or other resources? | 0 = Never 1 = Sometimes 2 = Often 99 = Refused to answer | 1 = Mild  2 = Moderate  3 = Severe |  |
|  | During the last 12 months, was there a time when you or others in your household ate less than you thought you should because of a lack of money or other resources? | 0 = Never 1 = Sometimes 2 = Often 99 = Refused to answer | 1 = Mild  2 = Moderate  3 = Severe |  |
|  | During the last 12 months, was there a time when you or others in your household were hungry but did not eat because there was not enough money or other resources for food? | 0 = Never 1 = Sometimes 2 = Often 99 = Refused to answer | 1 = Mild  2 = Moderate  3 = Severe |  |
|  | During the last 12 months, was there a time when you or others in your household were hungry but did not eat because there was not enough money or other resources for food? | 0 = Never 1 = Sometimes 2 = Often 99 = Refused to answer | 1 = Mild  2 = Moderate  3 = Severe |  |
|  | During the last 12 months, was there a time when you or others in your household went without eating for a whole day because of a lack of money or other resources? | 0 = Never 1 = Sometimes 2 = Often 99 = Refused to answer | 1 = Mild  2 = Moderate  3 = Severe |  |
| Psychological wellbeing (MHC-SF) | During the past month, how often did you feel happy? | 1 = Never  2 = Once or twice  3 = About once a week  4 = About 2 to 3 times a week  5 = Almost everyday  6 = Everyday | 0 = Flourishing [5,6]  1 = Moderately mentally healthy to languishing [1,2,3,4] |  |
|  | During the past month, how often did you feel interested in life? | 1 = Never  2 = Once or twice  3 = About once a week  4 = About 2 to 3 times a week  5 = Almost everyday  6 = Everyday | 0 = Flourishing [5,6]  1 = Moderately mentally healthy to languishing [1,2,3,4] |  |
|  | During the past month, how often did you feel satisfied? | 1 = Never  2 = Once or twice  3 = About once a week  4 = About 2 to 3 times a week  5 = Almost everyday  6 = Everyday | 0 = Flourishing [5,6]  1 = Moderately mentally healthy to languishing [1,2,3,4] |  |
|  | During the past month, how often did you feel that you had something important to contribute to society? | 1 = Never  2 = Once or twice  3 = About once a week  4 = About 2 to 3 times a week  5 = Almost everyday  6 = Everyday | 0 = Flourishing [5,6]  1 = Moderately mentally healthy to languishing [1,2,3,4] |  |
|  | During the past month, how often did you feel that you belonged to a community (like a social group or your neighborhood)? | 1 = Never  2 = Once or twice  3 = About once a week  4 = About 2 to 3 times a week  5 = Almost everyday  6 = Everyday | 0 = Flourishing [5,6]  1 = Moderately mentally healthy to languishing [1,2,3,4] |  |
|  | During the past month, how often did you feel that our society is becoming a better place for people like you? | 1 = Never  2 = Once or twice  3 = About once a week  4 = About 2 to 3 times a week  5 = Almost everyday  6 = Everyday | 0 = Flourishing [5,6]  1 = Moderately mentally healthy to languishing [1,2,3,4] |  |
|  | During the past month, how often did you feel that people are basically good? | 1 = Never  2 = Once or twice  3 = About once a week  4 = About 2 to 3 times a week  5 = Almost everyday  6 = Everyday | 0 = Flourishing [5,6]  1 = Moderately mentally healthy to languishing [1,2,3,4] |  |
|  | During the past month, how often did you feel that the way our society works makes sense to you? | 1 = Never  2 = Once or twice  3 = About once a week  4 = About 2 to 3 times a week  5 = Almost everyday  6 = Everyday | 0 = Flourishing [5,6]  1 = Moderately mentally healthy to languishing [1,2,3,4] |  |
|  | During the past month, how often did you feel that you like most parts of your personality | 1 = Never  2 = Once or twice  3 = About once a week  4 = About 2 to 3 times a week  5 = Almost everyday  6 = Everyday | 0 = Flourishing [5,6]  1 = Moderately mentally healthy to languishing [1,2,3,4] |  |
|  | During the past month, how often did you feel good at managing the responsibilities of your daily life? | 1 = Never  2 = Once or twice  3 = About once a week  4 = About 2 to 3 times a week  5 = Almost everyday  6 = Everyday | 0 = Flourishing [5,6]  1 = Moderately mentally healthy to languishing [1,2,3,4] |  |
|  | During the past month, how often did you feel that you had warm and trusting relationships with others? | 1 = Never  2 = Once or twice  3 = About once a week  4 = About 2 to 3 times a week  5 = Almost everyday  6 = Everyday | 0 = Flourishing [5,6]  1 = Moderately mentally healthy to languishing [1,2,3,4] |  |
|  | During the past month, how often did you feel that you had experiences that challenged you to grow and become a better person? | 1 = Never  2 = Once or twice  3 = About once a week  4 = About 2 to 3 times a week  5 = Almost everyday  6 = Everyday | 0 = Flourishing [5,6]  1 = Moderately mentally healthy to languishing [1,2,3,4] |  |
|  | During the past month, how often did you feel confident to think or express your own ideas and opinions? | 1 = Never  2 = Once or twice  3 = About once a week  4 = About 2 to 3 times a week  5 = Almost everyday  6 = Everyday | 0 = Flourishing [5,6]  1 = Moderately mentally healthy to languishing [1,2,3,4] |  |
|  | During the past month, how often did you feel that your life has a sense of direction or meaning to it? | 1 = Never  2 = Once or twice  3 = About once a week  4 = About 2 to 3 times a week  5 = Almost everyday  6 = Everyday | 0 = Flourishing [5,6]  1 = Moderately mentally healthy to languishing [1,2,3,4] |  |
| Depressive symptoms (CESD-10) | 10 item scale | 40 highest possible score | Presence of depressive symptoms:  <12 No depressive symptoms  >=12 Yes depressive symptoms |  |
|  | During the past week, I was bothered by things that usually don’t bother me. | 1 = Rarely or none of the time (less than 1 day) 2 = Some or little of the time (1-2 days) 3 = Occasionally or moderate amount of time (3-4 days) 4 = Most of or all of the time (5-7 days) | Yes >=12  No <12 |  |
|  | During the past week, I had trouble keeping my mind on what I was doing | 1 = Rarely or none of the time (less than 1 day) 2 = Some or little of the time (1-2 days) 3 = Occasionally or moderate amount of time (3-4 days) 4 = Most of or all of the time (5-7 days) | Yes >=12  No <12 |  |
|  | During the past week, I felt depressed | 1 = Rarely or none of the time (less than 1 day) 2 = Some or little of the time (1-2 days) 3 = Occasionally or moderate amount of time (3-4 days) 4 = Most of or all of the time (5-7 days) | Yes >=12  No <12 |  |
|  | During the past week, I felt that everything I did was an effort | 1 = Rarely or none of the time (less than 1 day) 2 = Some or little of the time (1-2 days) 3 = Occasionally or moderate amount of time (3-4 days) 4 = Most of or all of the time (5-7 days) | Yes >=12  No <12 |  |
|  | During the past week, I felt hopeful about the future | 1 = Rarely or none of the time (less than 1 day) 2 = Some or little of the time (1-2 days) 3 = Occasionally or moderate amount of time (3-4 days) 4 = Most of or all of the time (5-7 days) | Yes >=12  No <12 |  |
|  | During the past week, I felt fearful | 1 = Rarely or none of the time (less than 1 day) 2 = Some or little of the time (1-2 days) 3 = Occasionally or moderate amount of time (3-4 days) 4 = Most of or all of the time (5-7 days) | Yes >=12  No <12 |  |
|  | During the past week, my sleep was restless. | 1 = Rarely or none of the time (less than 1 day) 2 = Some or little of the time (1-2 days) 3 = Occasionally or moderate amount of time (3-4 days) 4 = Most of or all of the time (5-7 days) | Yes >=12  No <12 |  |
|  | During the past week, I was happy | 1 = Rarely or none of the time (less than 1 day) 2 = Some or little of the time (1-2 days) 3 = Occasionally or moderate amount of time (3-4 days) 4 = Most of or all of the time (5-7 days) | Yes >=12  No <12 |  |
|  | During the past week, I was lonely | 1 = Rarely or none of the time (less than 1 day) 2 = Some or little of the time (1-2 days) 3 = Occasionally or moderate amount of time (3-4 days) 4 = Most of or all of the time (5-7 days) | Yes >=12  No <12 |  |
|  | During the past week, I could not get going | 1 = Rarely or none of the time (less than 1 day) 2 = Some or little of the time (1-2 days) 3 = Occasionally or moderate amount of time (3-4 days) 4 = Most of or all of the time (5-7 days) | Yes >=12  No <12 |  |
| Caregiver burden (CarerQoL-7D) | 7 items | 21 = Highest possible score | High caregiver burden <= 7  Low caregiver burden |  |
| Caregiver satisfaction/ satisfaction (CarerQoL-VAS) | I have_____ fulfilment from carrying out my care tasks. | 1 = No  2 = Some  3 =A lot of | High >=7  Low <7 |  |
|  | I have _____ relational problems with the care receiver (e.g. he/she is very demanding or he/she behaves differently, we have communication problems) | 1 = No  2 = Some  3 =A lot of | High >=7  Low <7 |  |
|  | I have _____ problems with my own mental health e.g., stress, fear, gloominess, depression, concern about the future) | 1 = No  2 = Some  3 =A lot of | High >=7  Low <7 |  |
|  | I have ____ problems combining my care tasks with my daily activities (e.g., household activities, work, study, family and leisure activities) | 1 = No  2 = Some  3 =A lot of | High >=7  Low <7 |  |
|  | I have ____ financial problems because of my care tasks. | 1 = No  2 = Some  3 =A lot of | High >=7  Low <7 |  |
|  | I have ____ support with carrying out my care tasks when I need it (e.g. from family, friends, neighbours, acquaintances) | 1 = No  2 = Some  3 =A lot of | High >=7  Low <7 |  |
|  | I have ___ problems with my own physical health (more often sick, tiredness, physical stress) | 1 = No  2 = Some  3 =A lot of | High >=7  Low <7 |  |
|  | How happy do you feel at the moment? | Slider (number, max 10).  Slider labels: completely unhappy, completely happy | Low <5  High >=5 |  |
| Social grant recipient | Please indicate all the sources of income that your household receives. | 1 = Income from employment  2 = Income from business  3 = Government grants  4 = Pension  5 = Other  6 = N/A  99 = Refused | 1, 2, 4 & 5 = No  3 = Yes |  |
|  | Did you receive any government grant (or grants) do you receive on behalf of someone else | 0 = Yes  1 = No | 1 = Yes  2 = No |  |
|  | Do you personally receive any kind of government grant? | 0 = Yes  1 = No | 1 = Yes  2 = No |  |
| **Poverty threshold (R1417,00) | If you get some sort of income, can you please indicate how much you earn in a month? | Number (Rands) | 0 <= R1417 = Above poverty line  1 >R1417 = Below poverty line |  |

* Grade 9 is the minimal or compulsory level of education.

** Monthly upper bound poverty line as of 2022 (STATISTA ) [South Africa: national poverty line 2022 | Statista](https://www.statista.com/statistics/1127838/national-poverty-line-in-south-africa/)
